# Evaluation of Clinicopathological and Prognostic Factors of Male Breast Cancer: A Single-Centre Experience

**DOI:** 10.7759/cureus.20298

**Published:** 2021-12-09

**Authors:** Saroj Kumar Das Majumdar, Deepak K Das, Sandip Barik, Avinash Badajena, Dillip Kumar Parida

**Affiliations:** 1 Radiation Oncology, All India Institute of Medical Sciences, Bhubaneswar, IND

**Keywords:** india, invasive ductal carcinoma, clinicopathological, epidemiology, male breast cancer

## Abstract

Male breast cancer (MBC) is a rare malignancy with an overall incidence of less than 1%. The epidemiological data of MBC is very limited owing to its rarity, particularly data from India. Hence, it is important to study different aspects of this rare malignancy. This paper reports a single-center experience from India that evaluated the clinicopathological behavior of MBC, their management, and outcomes. This was a retrospective review, which included 18 patients managed between 2013 to 2021. Seventeen out of the 18 patients were aged ≥50 years; the median age was 60 years. Left sides were affected more than right (left: 11, right: seven), and the most commonly affected quadrant was central (n=15/17, 88.2%). The most common (n=14) surgery was modified radical mastectomy (MRM), and the invasive ductal carcinoma was the most common (n=14) histological finding. Most cases were estrogen-receptor (ER) and progesterone-receptor-positive (n=15/18, 83.3%). The present study, though with a small sample size, adds valuable information to the literature about this rare occurrence in men.

## Introduction

Male breast cancer (MBC) is a rare malignancy with an overall incidence of less than 1% [1,2]. In India, there is limited data on the MBC. A study from New Delhi has shown an age-adjusted incidence rate of 0.3 per 100,000 population [3]. The risk of MBC increases with age, and most of the patients consult at a very advanced stage because of a higher chance of skin and chest wall involvement due to scarcity of breast tissue in males. Several factors have been considered to increase the risk of breast cancer in males, including positive family history, age, Klinefelter syndrome, history of estrogen or testosterone use, obesity, primary testicular disorders, and history of radiation exposure to the thoracic region [4,5]. Around 15% to 20% of MBC patients have a positive family history [6,7].

The epidemiological data of MBC is very limited owing to its rarity, particularly data from India; however, the incidence of MBC is slowly increasing and being reported widely [8]. Hence, it is important to study different aspects of this rare malignancy. This paper reports a single-center experience from India that evaluated the clinicopathological behavior of MBC, their management, and outcomes.

## Materials and methods

This was a retrospective review of all male breast cancer cases managed at a tertiary care institute from 2013 to 2021. All patients diagnosed with male breast cancer were identified from the hospital record and data was collected to note age, comorbidities, side, quadrant, and duration of the symptom; tumor, node, and metastasis (TNM) classification [American Joint Committee on Cancer (AJCC) 8^th^ edition], treatment (including the type of surgery and date), histopathological parameters and details of relapse and its management. Data were collected and evaluated using descriptive statistics.

## Results

A total of 18 patients with male breast cancer were managed at our hospital. Of these, 17 out of 18 patients (94.4%) were aged ≥50 years; the mean age was 60.27 years (median: 60 years; range: 48 to 72 years) (Table 1). Four patients had a history of diabetes mellitus, and three of these patients also had hypertension. Left sides were more affected than right (left-11, right-7), and the most commonly affected quadrant was central (n=15/17, 88.2%). The median duration of the lesion before diagnosis was three months (range: one to 24 months). One patient had a family history of breast cancer (Three sisters were affected before age 50).

On clinical and radiological evaluation, 14 patients (77.7%) presented with stage III/IV disease. Six patients had metastasis at presentation (Table 1). Bone metastasis, lung metastasis, and multiple site metastasis were seen in three, one, and two patients, respectively. Two patients with bone metastasis had a single lesion with increased osteoblastic activity on bone scan (oligometastasis).

**Table 1:** Clinicopathological parameters

Clinicopathological parameters		No. of patients (n=18)
Median age		60 years (48-72)
Comorbidity		Diabetes mellitus: 3, hypertension: 3
Family history		1
Median duration of presentation before diagnosis		3 months (1 to 24 months)
Laterality		Left: 11, Right: 7
Quadrant		Central: 15/17, Data of one patient unavailable
Stage	I	1
	II	3
	III	8
	IV	6 (Bone: 3, Lungs: 1, Multiple site: 2)
Histology	IDC	15
	Carcinoma NOS	1
	Apocrine Carcinoma	1
	Syringocystadenocarcinoma	1
Hormonal Status	ER/PR	15
	HER2NEU	6
	Triple negative	1

A total of nine patients received neoadjuvant chemotherapy (NACT). The most common (n=14) surgery was modified radical mastectomy (MRM) (Table 2). One out of nine patients receiving NACT had a pathological complete response. The histopathological reports showed that around 15 of 18 patients (83.3%) had invasive ductal carcinoma (IDC), and the other three patients had apocrine carcinoma, Syringo cystadenocarcinoma, and Carcinoma NOS, respectively (n=1, each). Both ER and PR were positive in 15 out of 18 patients (83.3%) by immunohistochemistry (IHC). Six patients had Her2neu receptor positivity by IHC (33.3%). ER, PR and Her2neu receptors were negative (triple-negative) in one patient. Pathological positive nodes were found in six patients. Extracapsular extensions were found in three out of six pathologically lymph-node-positive patients. Margins were negative in all postoperative patients. Lymphovascular emboli (LVE) was positive in seven of 14 patients, and perineural invasion (PNI) was positive in five out of 14 patients (Table 2). Breast Cancer (BRCA) 1 and 2 mutation analysis was done for one patient with a family history of breast cancer, and it was found to be negative.

**Table 2:** Treatment and outcome parameters

Parameters		No of patients
Surgery performed		14
Post op histopathology	LN involvement	6
	Extracapsular extension	3
	Lymphovascular invasion	7
	Perineural invasion	5
Neoadjuvant chemotherapy		9
Adjuvant chemotherapy		11
Adjuvant radiotherapy		9
Hormonal therapy	Tamoxifen	10
	Letrozole	2
	Anastrozole	1
Status at last follow up	Clinical no evidence of disease	12
	Alive with residual disease	3
	Dead	1 (Two patients did not turn up for treatment after diagnosis)

Treatment details of 16 out of 18 patients were available (Table 2). Two patients did not turn up for treatment after complete staging workup. After a median follow-up of 35 months (range: one day to 106 months), the majority of the patients (75%; n=12) had no evidence of disease. Three patients had residual disease. One patient was dead at the last follow-up who presented with stage IV disease and second malignancy of rectal non-Hodgkins lymphoma. One patient had three relapses. The first relapse was after 23 months of the surgery without receiving any form of adjuvant treatment, where the patient had metastases in the supraclavicular fossa, chest wall, and lungs. The patient was treated with six cycles of doxorubicin and epirubicin followed by tamoxifen. This patient had a second relapse (metastases to lungs) after 15 months of the first relapse and was treated with six cycles of paclitaxel and cyclophosphamide, followed by cyclophosphamide, anthracycline, and then received tamoxifen for maintenance. Again after 32 months of the second relapse, the patient had a third relapse (supraclavicular fossa, September 2018) and received letrozole. At the last follow-up, he was found to have progressive disease with chest wall nodules and increased size of the supraclavicular node.

Disease-free survival (DFS) was calculated from the date of surgery to the day of the study. DFS data was available for nine patients only. Median DFS was 51 months (19-86 months).

## Discussion

The MBC is a rare malignancy, and there is limited data available from India [4, 9-17]. Studies have reported the prevalence of MBC <0.5% in India [13-15]. MBC is generally seen in the elderly population aged > 60 years; however, few reports have shown its occurrence in younger patients as well [9]. Previously, a case of a 14-year-old boy was reported from India [18]; however, Hartman and Magrish have reported a case of a six-year-boy [19]. The mean age at diagnosis for men with breast cancer is 67 years, which is five years older than the average age at diagnosis for women [20]. In the present study, the median age was 60 years, and patients' age ranged from 48 to 72 years. Several factors are considered to be contributing to or increasing the risk of breast cancer, which includes hormonal imbalances, obesity, testicular disorders, and radiation exposure, prostate cancer, gynecomastia, occupational exposures.

A family history of breast cancer is considered to be associated with the increased risk of MBC. In the present study, we found only one patient with a family history of breast cancer. Mutation in the BRCA1/2 gene increases the risk of breast cancer in men but not in females. In a series of high-risk families, 10%-16% of men with breast cancer are found to have BRCA1 mutation [20]. BRCA1/2 mutation was not found in one of our patients with a family history of breast cancer. In the present study, the majority of histopathological reports showed IDC, which was also consistent with previous reports [4,12,16]. A previous retrospective study by Sundriyal et al. reported 18 patients of MBC during January 2005 to December 2014 where the age ranged from 42 years to 70 years, and all patients had IDC [11]. Most of the patients presented in the advanced stage (11 patients had distant metastasis) [11]. 

In another retrospective study, 53 MBC were evaluated from January 2005 to December 2015, of which a positive family history of breast cancer was noted in 7.5% of patients, history of testicular trauma in 5.6%, obesity was noted in 11.3%, and sedentary lifestyle in 32% of patients [4]. Another study by Ram et al. evaluated 27 MBC patients retrospectively, who visited between January 2010 to April 2016, and the mean age was 62.6 years [12]. Of the total 27 patients, only two patients had a family history of male breast cancer.

In our study, left sides were affected more, but among quadrants, the central quadrant was most common. In the previous studies, the central quadrant was affected in 58.8% [12] and 43.3% [4].

In our study, 14 patients (87.5%) had MRM, and nine patients (56.2%) also received neoadjuvant chemotherapy. Eleven patients (68.7%) received adjuvant chemotherapy, and nine patients (56.2%) received adjuvant radiotherapy. Thirteen patients (81.2%) received hormonal therapy, out of which 10 patients received tamoxifen (76.9%). In a previous study by Sundriyal et al., 38.89% were managed with surgery and 61.11% with chemotherapy [11]. Andleeb et al. used MRM in 75.4% patients 13.2 had a lumpectomy with the axillary sample; however, 69.8% of patients received adjuvant chemotherapy, and 79.25% patients received radiotherapy [4]. In another study by Ram et al., 70.4% received adjuvant chemotherapy, and 22.2% received adjuvant radiotherapy [12]. The survival rates vary in different reports with a range from 40%, five-year disease-free survival, to 92% of overall five-year survival [9,10,12,15,17]. In our study after a median follow-up of 35 months (one day to 106 months), 15 patients (93.7%) were alive, out of which 12 patients had no clinical evidence of disease. Median DFS was 51 months.

## Conclusions

The available literature suggests that a majority of the data on MBC is from retrospective reports. Most of the treatment recommendations are extrapolated from that of female counterparts. The present study, though with small sample size, adds valuable information to the literature about this unusual occurrence in men.
